# The impact of mangiferin from *Belamcanda chinensis* on experimental colitis in rats

**DOI:** 10.1007/s10787-017-0337-0

**Published:** 2017-03-24

**Authors:** Marta Szandruk, Anna Merwid-Ląd, Adam Szeląg

**Affiliations:** Department of Pharmacology, Wroclaw Medical University, Mikulicza-Radeckiego 2, 50-345 Wrocław, Poland

**Keywords:** Inflammatory bowel disease, Experimental colitis, Trinitrobenzensulfonic acid, Mangiferin, Tumor necrosis factor α, Interleukin-17

## Abstract

**Background:**

Inflammatory bowel disease (IBD) [including Crohn’s disease (CD) and ulcerative colitis (UC)] constitutes an important clinical problem. The pathogenesis of IBD remains unclear. It is believed that immune dysfunction, inflammatory mediators and oxidative damage play crucial roles in development of IBD. The condition is clinically associated with symptoms ranging from mild to severe during relapses, depending on the affected segment of the gastrointestinal tract. Bloody diarrhea with mucus, abdominal pain, weight loss and anemia are initial symptoms of both CD and UC. Differences between diseases become more evident in time, along with the development of intestinal and extraintestinal complications. Mangiferin (1,3,6,7-tetrahydroxyxanthone-C-2-β-d-glucoside), a natural polyphenol in plants, exerts antioxidant and anti-inflammatory effects making it an interesting option for the treatment of inflammatory pathologies associated with oxidative stress in humans, such as IBD.

**Purpose:**

The aim of the current study was to elucidate the impact of mangiferin on colon tissues in 2,4,6-trinitrobenzensulfonic acid (TNBS)-induced colitis in rats.

**Methods:**

Mangiferin was obtained from *Belamcanda chinensis* rhizomes by a multistage process. Groups of rats were pre-treated with 10, 30 or 100 mg/kg of mangiferin, or with distilled water administered intragastrically for 16 days. An ethanol solution of TNBS or saline was given rectally on the day 15 of the experiment. The experiment was terminated on the day 17. The colon was removed, cleaned, weighed and examined macro- and microscopically. Determination of tumor necrosis factor α (TNF-α), interleukin 17 (IL-17), malondialdehyde (MDA) levels and superoxide dismutase (SOD) activity were performed spectrophotometrically in homogenates of colon tissues.

**Results:**

Rats in the TNBS group developed symptoms of colitis, including: body weight loss, colon mass index increase and damage of intestinal tissues with concomitant increase in TNF-α, IL-17, MDA levels and decreased SOD activity. In non-TNBS-treated rats mangiferin did not cause any changes of studied parameters. Pre-treatment with mangiferin exerted a protective effect, reducing the intensity of damage caused by TNBS. Mangiferin at the doses of 30 and 100 mg/kg reduced the macro- and microscopic damage score and the MDA level in colon tissues. Only at the dose of 100 mg/kg, mangiferin decreased TNF-α and IL-17 concentrations, and SOD activity in colon tissues.

**Conclusion:**

Mangiferin attenuates inflammatory changes of colon tissues in experimental, TNBS-induced colitis in rats. Protective effect exerted by mangiferin depends primarily on its anti-inflammatory activity and secondarily on its antioxidant properties.

## Introduction

Inflammatory bowel disease (IBD) is an important and commonly encountered clinical problem. The incidence of IBD has considerably increased in recent years in both Western and Eastern societies, also in children. Almost 25% of new diagnoses of the disease are made in patients under 18 years of age. The most common forms of IBD are Crohn’s disease (CD) and ulcerative colitis (UC). IBD is characterized by a chronic, relapsing inflammatory condition of the gastrointestinal tract, with both overlapping and distinct pathological and clinical features. Symptoms range from mild to severe during relapses and may disappear during remissions, depending on the involved segment of the gastrointestinal tract. Initially, both CD and UC are characterized by low-grade fever, fatigue, bloody diarrhea accompanied by mucorrhea, abdominal pain with cramps, unintended weight loss with reduced appetite and anemia. Differences between Crohn’s disease and ulcerative colitis patients become more evident with progression of the disease, along with development of intestinal and extraintestinal complications (Sartor 2006; Vasovic et al. 2016).

The pathogenesis of IBD is multifactorial and remains not completely understood. It is presumed that a complex interaction between genetic, environmental or microbial factors may lead to enhanced immune response, involving increased migration, proliferation and activation of Th1, Th2 and Th17 cells and increased concentrations of pro-inflammatory cytokines, including tumor necrosis factor α (TNF-α) and interleukin 17 (IL-17), that determine the development and persistence of inflammation. Moreover, oxidative stress and a loss of mucosal epithelial barrier may contribute to the development of intestinal inflammation (Pavlick et al. 2002; Sartor 2006; Shen and Durum 2010).

Despite a significant progress in IBD therapy, clinicians still expect more effective and safer medicines. Presently, aminosalicylates are considered drugs of choice for the management of majority of cases, while glucocorticosteroids, immunosuppressants and biological agents are reserved for more severe forms of the disease (van Assche et al. 2010; Dignass et al. 2012). The pharmacological treatment is fraught with severe side effects of drugs. Those effects are particularly important in the context of chronic and relapsing nature of IBD and the necessity of long-term pharmacotherapy (Kenneth and McQuaid 2012). Thus, there is a need for identification of new, more efficient and safer therapies for IBD. The assessment of substances obtained from plants, traditionally used in various inflammatory conditions, is one of the approaches for development of future IBD treatments.

Mangiferin (2-C-β-d-glucopyranosyl-1,3,6,7-tetrahydroxyxanthone) belongs to xanthonoids, a class of natural components referred to as polyphenols. Mangiferin is mainly isolated from leaves, bark and fruit peel of *Mangifera indica* (L.) and rhizomes of *Belamcanda chinensis* (L.) DC. (syn.: *Iris domestica* (L.) Goldbl. & Mabb.; English: blackberry lilly, leopard flower; Traditional Chinese Medicine: *she gan*). These rhizomes are used in East Asian phytotherapy systems as an antipyretic, antiphlogistic, analgesic and expectorant agent. Studies with mangiferin based on experimental in vitro and in vivo assays have demonstrated its antioxidant, anti-inflammatory, immunomodulatory, antimicrobial, analgesic and radioprotective effects (Matkowski et al. 2013; Wauthoz et al. 2007).

This study was designed to elucidate the impact of mangiferin on 2,4,6-trinitrobenzensulfonic (TNBS) acid-induced colitis in rats.

## Materials and methods

### Plant material

**Fig. 1:**
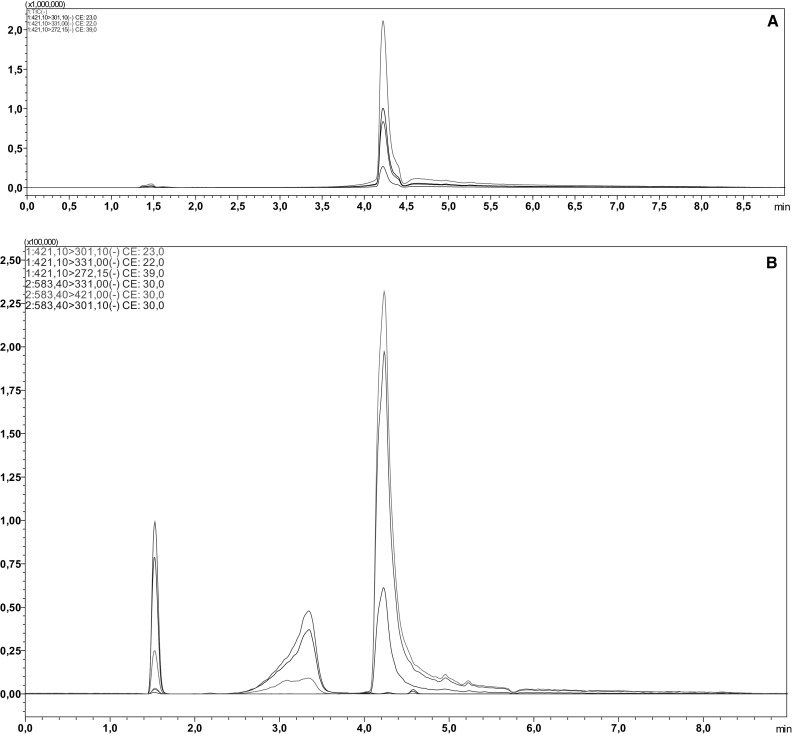
Multiple reaction monitoring (MRM) chromatograms recorded during the analysis of the methanol extract of mangiferin standard (**a**) and obtained mangiferin (**b**). In the whole range of peak there is only one mass 421.1 m/z

Mangiferin was isolated from *Belamcanda chinensis* rhizomes obtained from the Botanical Garden of the Faculty of Pharmacy, Wroclaw Medical University, Wroclaw, Poland. A voucher specimen was deposited in the Department of Pharmaceutical Biology and Botany of the Faculty of Pharmacy, Wroclaw Medical University, Wroclaw, Poland (Voucher No. 24.2014). First, rhizomes were dried and powdered. Crude material was defatted with hexane and extracted with methanol for 24 h. The methanol extract was evaporated, suspended in 20% methanol and partitioned with diethyl ether followed by butanol. The butanol extract was subjected to solid-phase extraction on C-18 silica cartridges eluted with water and subsequently with 40% methanol. The 40% methanol fraction was further separated with reversed-phase flash chromatography using C-18 silica as the stationary phase eluted with 1% acetic acid and 25% acetonitrile under 0.3 Bar pressure of nitrogen. Mangiferin was eluted as the first fraction. Pure mangiferin was obtained after recrystallization from ethanol and drying under nitrogen. Liquid chromatography-mass spectrometry (Fig. 1) and quantitative analysis were carried out, respectively, to confirm the chemical identity and purity of obtained mangiferin.

### Animals

Male Wistar rats (160–220 g) were purchased from the Department of Pathomorphology of the Wroclaw Medical University (Wroclaw, Poland). All rats were housed, two per cage, in polypropylene cages with water ad libitum. The rats were kept in controlled environment (temperature of 21–24 °C and 55–60% humidity) with a 12 h light/dark cycle with free access to standard rodent chow (Agropol, Poland) except for the single procedure of deprivation. The study was approved by the First Local Ethics Committee for Experiments on Animals in Wroclaw (No. 18/2013).

### Chemicals

Chemicals used in the experiment were as follows: trinitrobenzensulfonic acid (Sigma Aldrich, Germany); pentobarbital sodium (Biowet, Poland); physiological saline (Polpharma S.A., Poland); ethanol and formaldehyde (Avantor, Poland); acetic acid, methanol, butanol, diethyl ether, hexane (Chempur, Poland); acetonitrile (Merck, Germany) and C-18 silica gel (Sigma Aldrich, Germany).

### Design of the experiment

After 2 weeks of adaptation, 79 rats were randomly divided into eight groups (9–10 rats in each) as follows: one group receiving distilled water intragastrically (i.g.) and once saline rectally (control group, K); three groups of rats receiving mangiferin at doses of 10, 30 or 100 mg/kg i.g. and once saline rectally (M_10_, M_30_, M_100_, respectively), one group receiving distilled water i.g. and once TNBS solution rectally (colitis group, C), and three groups receiving mangiferin at doses of 10, 30 or 100 mg/kg i.g. and a single rectal administration of TNBS solution (CM_10_, CM_30_, CM_100_, respectively). Because of very limited data concerning pure mangiferin doses in rat experimental models (mainly in other then IBD conditions) we have decided to use the dose-dependent model of the experiment with three doses of mangiferin. Distilled water or aqueous solution of mangiferin was given once daily by gastric tube (4 ml/kg b.w.) for 16 consecutive days. 0.3 ml of TNBS ethanol solution or saline was given rectally on day 15 of the experiment, after 24-h of food deprivation. Animals were observed and weighted every day. Forty-eight hours after induction of colitis rats were killed by cervical dislocation under deep pentobarbital anesthesia (53.4 mg/kg). The distal 8 cm segment of the colon was collected from each animal, placed on ice-cold plate and longitudinally opened, cleaned, weighed and macroscopically examined. Afterwards samples were divided into two pieces. One piece was fixed in 4% buffered formaldehyde for histopathological examination. The histological sections were from paraffin embedded tissues and they were stained using three methods: hematoxylin-eosin, Giemsa and alcian blue. The second piece was homogenized in phosphate-buffered saline solution (pH 7.4) and centrifuged at 40,000 rpm for 10 min at 4 °C. Obtained supernatants were transferred into new tubes and stored at −80 °C for biochemical analyses.

### Induction of colitis

Colonic inflammation was induced according to the procedure originally described by Morris et al. (1989). Briefly, rats were anesthetized with intramuscular injection of ketamine (75 mg/kg) and medetomidine (0.5 mg/kg) and were positioned on their right side. Then TNBS (50 mg/kg) dissolved in 50% ethanol (v/v) was instilled into the colon using a flexible polyethylene catheter (external diameter 2 mm) and inserted 8 cm deep into the rectum. Following the instillation of TNBS, animals were kept in Trendelenburg position for 5 min to prevent leakage of the instilled solution.

### Assessment of body weight and colon mass index

The difference of body weight between days 15 and 17 of the experiment was determined. The colon mass index (the ratio of excised 8 cm of colon weight to total body weight) was calculated.

### Assessment of macro- and microscopic damage of the colon

**Fig. 2:**
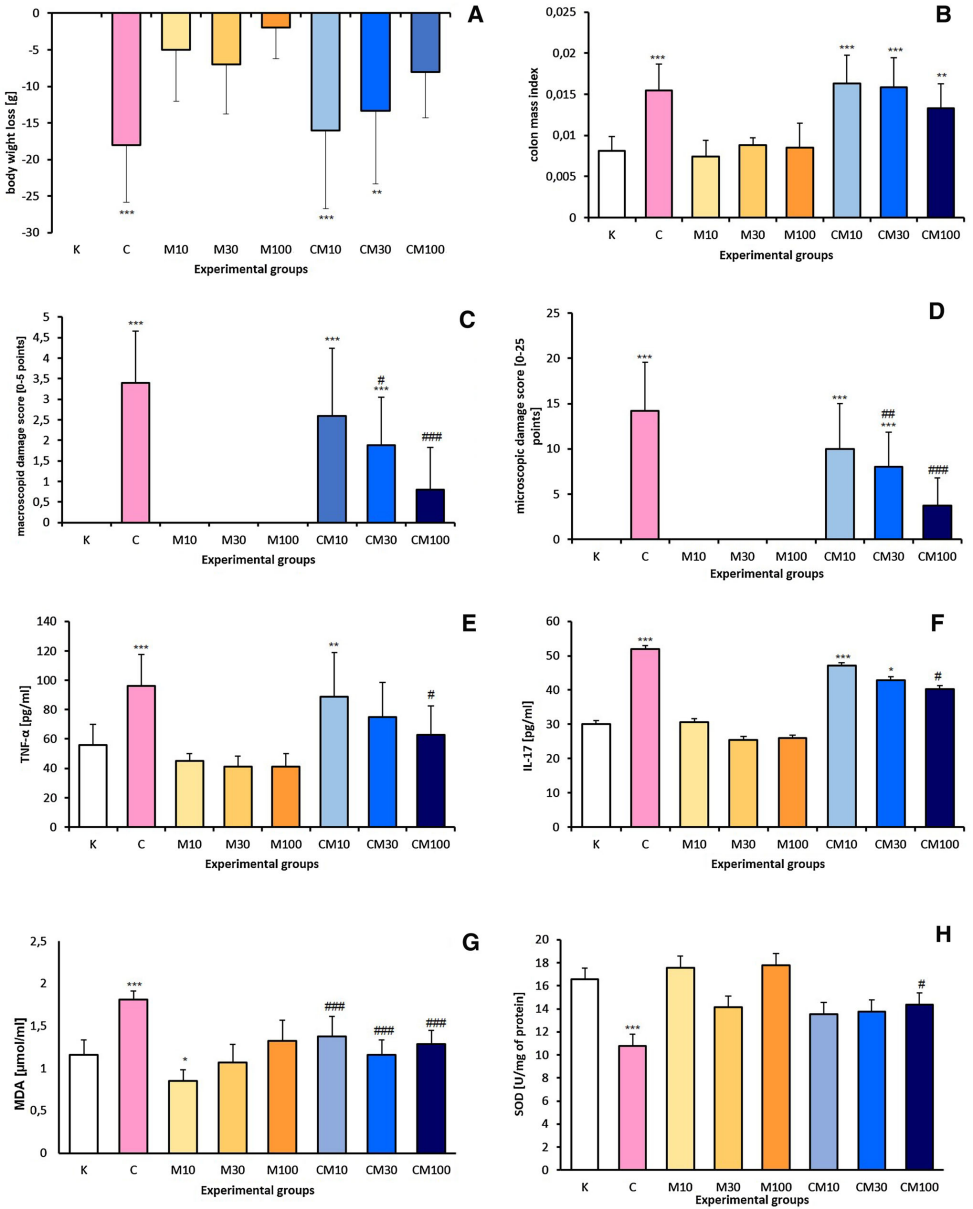
The impact of mangiferin on rat: body weight (**a**), colon mass index (**b**), macroscopic damage of colon tissues (**c**), microscopic damage of colon tissues (**d**), colon tissues TNF-α concentration (**e**), colon tissues IL-17 concentration (**f**), colon tissues MDA concentration (**g**), SOD activity in colon tissues (**h**) in experimental groups; K—the control group, M_10_, M_30_, M_100_—groups receiving, respectively, 10, 30 or 100 mg/kg of mangiferin intragastrically, C—the group receiving only TNBS rectally, CM_10_, CM_30_, CM_100_—groups receiving 10, 30 or 100 mg/kg of mangiferin with TNBS, respectively. Macroscopic evaluation of colonic tissue damage according to the criteria described by Galvez et al.: score 0 (no damage); score 1 (hyperemia, no ulcers); score 2 (linear ulcer with no significant inflammation); score 3 (linear ulcer with inflammation at one site); score 4 (two or more sites of ulceration or inflammation and ulceration or inflammation extending <1 cm); score 5 (two or more major sites of ulceration or inflammation extending >1 cm along the length of the colon) Microscopic evaluation of colonic tissue damage: in mucosal epithelium and lamina propria: ulceration (0–4), mononuclear cell infiltration (0–3), polymorphonuclear cell infiltration (0–3); in submucosa: edema (0–3), mononuclear cell infiltration (0–3), polymorphonuclear cell infiltration (0–3); in muscular layer: mononuclear cell infiltration (0–3), polymorphonuclear cell infiltration (0–3). Scoring scale: 0—none, 1—mild, 2—moderate, 3—severe, 4—full-thickness; maximum score: 25. Results are presented as mean values ± SD. Differences ***p < 0.001 vs the control group; **p < 0.01 vs the control group; *p < 0.05 vs the control group; ^###^ *p* < 0.001 vs the TNBS group; ^##^ *p* < 0.01 vs the TNBS group; ^#^ *p* < 0.05 vs the TNBS group were deemed statistically significant

The severity of colon tissue damage was measured using macro- and microscopic examination. The colon was examined visually immediately after resection. The severity of macroscopically visible changes in the mucous membrane was scored using the 0–5 scale according to the criteria described by Gálvez et al. (2001). Colonic specimens demonstrating macroscopic injury of the greater extent collected from each rat exposed to TNBS, or equivalent colonic specimens from non-colitic rats, were selected for the histological assessment. Selected specimens were fixed in 4% buffered formaldehyde and embedded in paraffin. Thereafter, tissues samples were cut into 4 µm-thick slices using a rotary microtome (Sakura Accu-cut SRM, Netherlands) and placed on glass slides. Preparations were stained using three methods: hematoxylin-eosin, Giemsa and alcian blue for histological assessment of colonic damage, cell infiltration and mucus content, respectively. Histological damage was evaluated using the scoring scale described in the legend to Fig. 2, based on the criteria previously presented by Arribas et al. (2010). The final result was the sum of points (0–25) scored by evaluation of each criterion. All slides were coded to prevent observer bias during the assessment. For characterization of histopathological changes all tissue sections were examined by two independent pathologist using the Olympus BX51 microscope, based on photographs taken from colon samples made with the Olympus DP72 digital camera. The assessment was carried out based on the image analysis system Olympus Soft Imaging Solutions GmbH—cell^D ver. 3.2. (Olympus, Germany).

### The assessment of TNF-α and IL-17 levels in colon tissues

TNF-α and IL-17 concentrations were assayed in obtained supernatants with quantitative enzyme immunoassay rat specific kits: Rat TNF-α Elisa Kit and Rat IL-17 Elisa Kit, respectively, (Diaclone, Francja) according to the manufacturer’s instructions. Concentrations of TNF-α and IL-17 were expressed as pg/ml.

### Determination of the MDA level and SOD activity in colon tissues

Both parameters were assessed spectrophotometrically (Marcel S350 Pro spectrophotometer, Poland) in obtained supernatants using assay kits according to their manufacturers’ instructions. Malondialdehyde (MDA) concentration was assayed using the Bioxytech-MDA-586 Kit (OxisResearch, USA) and its level was expressed as µmol/ml. Superoxide dismutase (SOD) activity was analyzed using the Ransod Kit (Randox Laboratories, UK). Values of SOD activity were expressed as U/mg protein. Total protein concentration in homogenates was assayed in commercial, certified laboratory using the enzymatic method.

### Statistical analysis

All data are presented as mean values ± standard deviation (SD). Statistical differences between studied parameters were analyzed using one-way analysis of variance (ANOVA) and multiple comparisons with the Tukey’s post hoc test. The relationship between studied parameters was evaluated by the Pearson correlation coefficient. All statistical analyses were performed with GraphPad Prism version 5.0 (GraphPad Software, USA), with statistical significance set at *p* < 0.05.

## Results

**Fig. 3:**
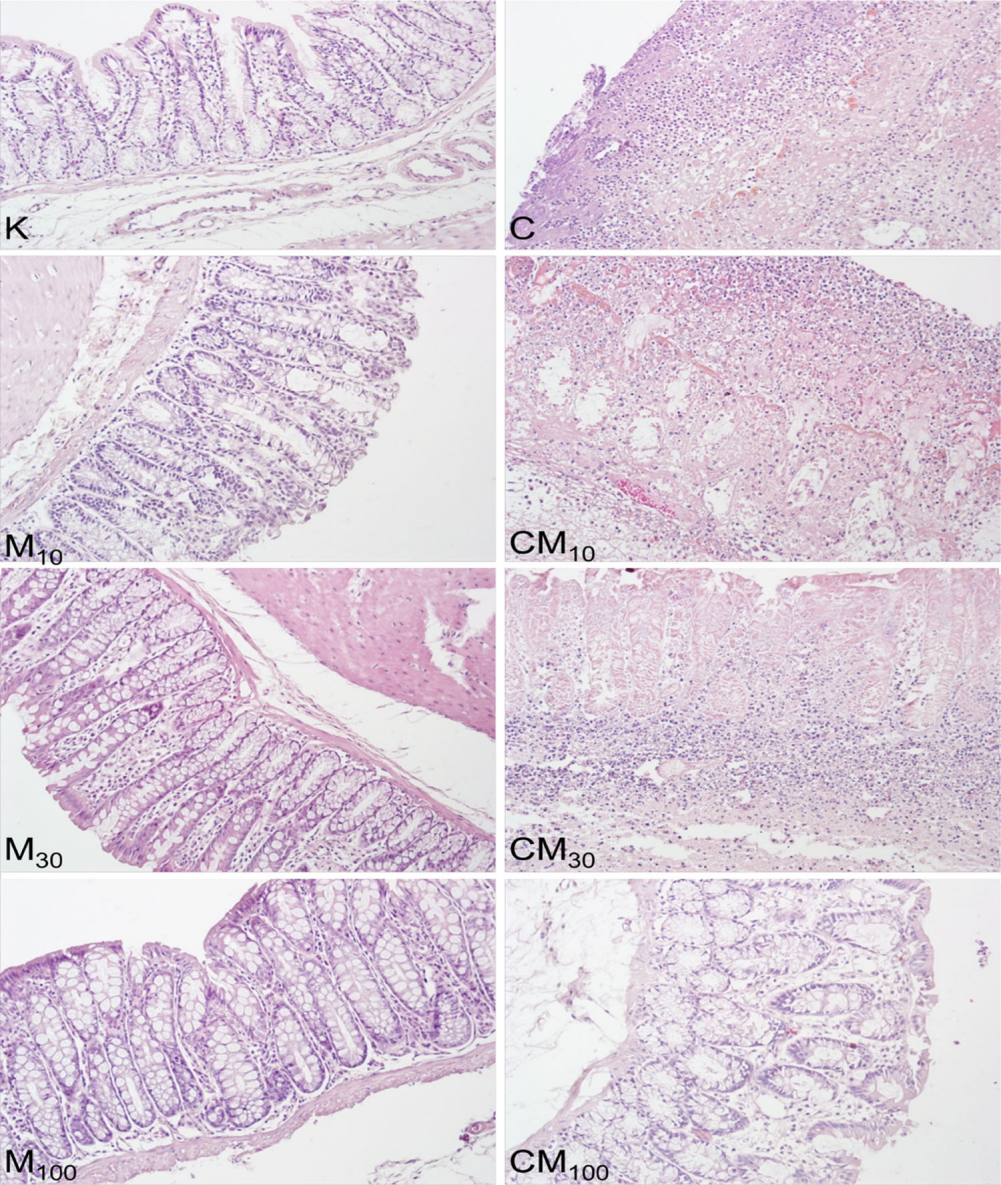
Microscopic appearance of colon tissues after hematoxylin-eosin staining showed that mangiferin reduces histological damage; control group (K), groups receiving mangiferin at the doses of 10 or 30 or 100 mg/kg (M_10_, M_30_, M_100_, respectively), group receiving only TNBS (C), groups receiving mangiferin at the doses of 10 or 30 or 100 mg/kg with TNBS (CM_10_, CM_30_, CM_100_, respectively); magnification 200×

**Fig. 4:**
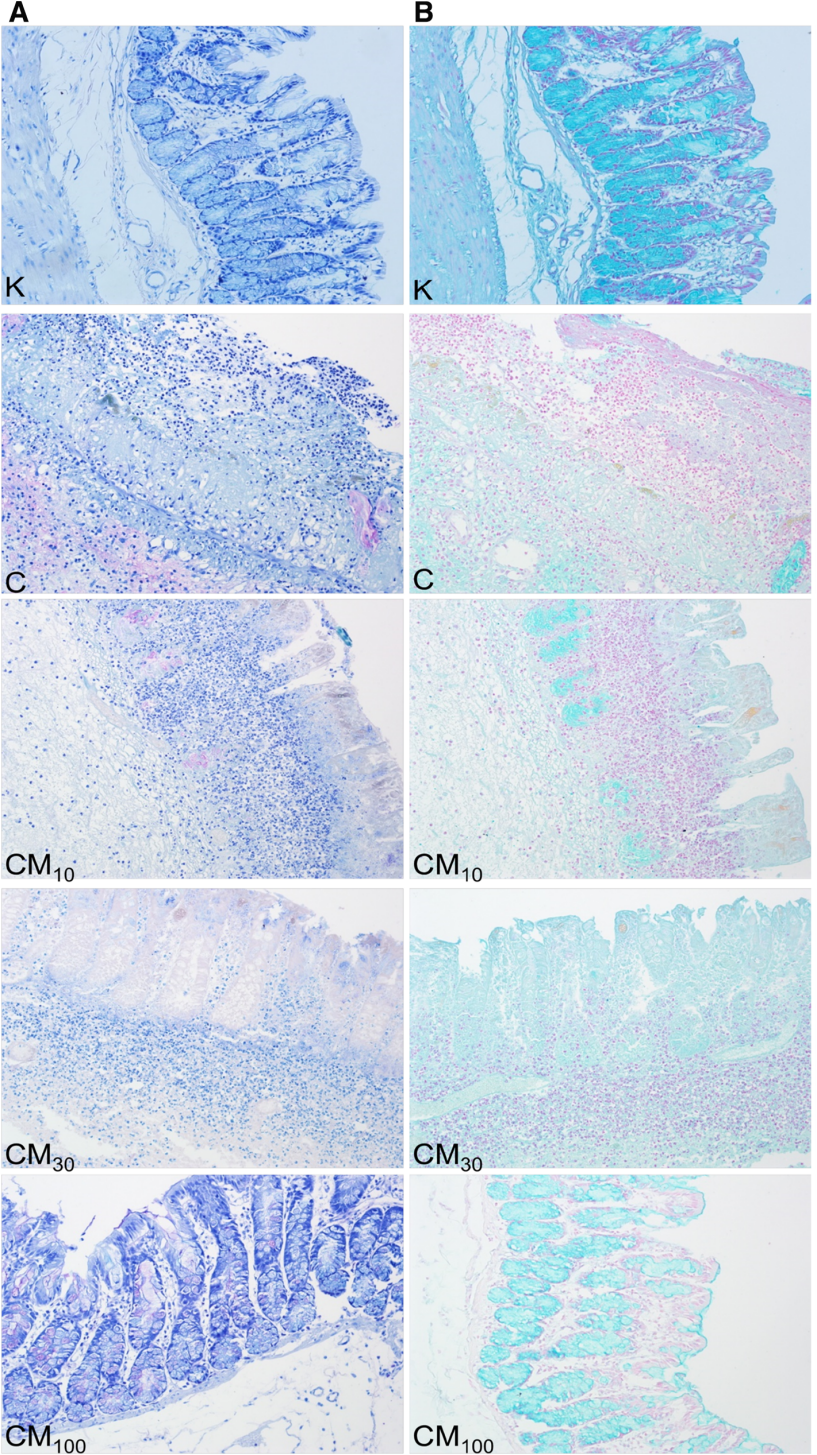
Microscopic appearance of colon tissues after Giemsa staining showed that mangiferin reduces the inflammatory cells infiltration (**a**); and after Alcian blue staining showed that mangiferin prevents loss of mucus layer (**b**); control group (K), group receiving only TNBS (C), groups receiving mangiferin at the doses of 10 or 30 or 100 mg/kg with TNBS (CM_10_, CM_30_, CM_100_, respectively); magnification 200×

Detailed effects (including the level of significance) of mangiferin on body weight, colon mass index, macro- and microscopic damages, TNF-α and IL-17 and MDA levels and SOD activity in colon tissues are presented in the Fig. 2. Summing up briefly, TNBS caused significant loss of body weight, increased colon mass index, elicited macro- and microscopic damages in colon tissues, increased TNF-α, IL-17 and MDA concentrations and decreased SOD activity, in comparison to the control group. Administration of mangiferin at the highest dose (100 mg/kg) significantly protected from TNBS-induced changes, such as macro- and microscopic damage, TNF-α, IL-17, MDA concentration increase and increased SOD activity, but not from TNBS-induced body weight loss and increased colon mass index. Mangiferin administered at the medium dose (30 mg/kg) protected only from macro- and microscopic damage and MDA level increase. The lowest dose of mangiferin (10 mg/kg) did not reverse any of the TNBS-induced changes. Administration of mangiferin in rats non-treated with TNBS did not exert any changes in any of studied parameters in comparison to the control group. Macroscopic changes of TNBS-induced colitis involved bowel wall thickening, hyperemia and edema. Microscopic examination of colon samples showed an intense interruption of colon tissues with ulceration and inflammation involving not only the epithelial layer but also all intestinal layers in some of studied specimens. The ulceration was associated with transmural infiltration of lymphocytes, macrophages and neutrophils. The mucosal infiltration of inflammatory cells was considerably high and it was minor in the submucosa and muscular layer. Rats with TNBS-induced colitis also showed structural distortion of crypts, desquamated areas or loss of epithelium. The inflammatory process was also linked with goblet cell depletion leading to the decline of the mucus layer of the epithelium (Fig. 3, 4). In comparison with the TNBS group mangiferin pre-treatment (30 or 100 mg/kg) before the induction of colitis significantly attenuated the colonic lesions and resulted in reduced score of macro- and microscopic damage. A histological examination of colonic samples from rats pre-treated with mangiferin (30 or 100 mg/kg) demonstrated a pronounced recovery of tissues of the colon, with decreased extent and reduced intensity of ulceration (Fig. 3), decreased transmural infiltration of inflammatory cells (Fig. 4a), increased amount of goblet cells refilled with their mucin constituents and restored epithelial cell layer (Fig. 4b). Mangiferin at the lowest dose (10 mg/kg) had no protective effect against TNBS-induced changes. The action of mangiferin on TNBS-induced colonic damage was dose dependent. It was significantly higher at the dose of 100 mg/kg compared to 30 mg/kg. Administration of mangiferin to rats non-exposed to TNBS did not exert any pathological changes of all studied parameters. Correlation coefficients between the microscopically evaluated degree damage of colonic tissue and TNF-α, IL-17, MDA concentrations (*r* = 0.692; 0.561; 0.663, respectively) and the SOD activity (*r* = −0.804) were statistically significant at *p* < 0.001. There was no directly proportional correlation between the score of microscopic damage and the above-mentioned parameters, but it has been demonstrated that the degree of microscopic damage done to the colon decreased along with decreasing TNF-α, IL-17 and MDA concentrations and increasing SOD activity.

## Discussion

Experimental animal models of IBD are valuable tools widely used in preclinical studies. In our study the TNBS model of IBD had been chosen because it is well characterized and shares some biochemical and immunological features and symptoms with the disease in humans (Westbrook et al. 2010).

Some efforts have been recently made to identify and extract purely natural compounds, particularly plant-derived ones, that could restore the normal immune response altered in IBD and act as protective agents in various inflammatory disorders. Polyphenols, represented by mangiferin, are one of the most important groups of natural substances, demonstrating strong anti-inflammatory and antioxidative properties (Márquez et al. 2010). Mangiferin has been widely evaluated in various in vitro assays that suggested its protective effect against oxidative damage and development of inflammation in tissues (Matkowski et al. 2013; Wauthoz et al. 2007). Our study was aimed at determination of the impact of mangiferin extracted from *Belamcanda chinensis* rhizome on the model of experimental colitis induced by rectal TNBS administration.

Based on results of the present study mangiferin did not exert any toxic effect on colon tissues. In the group of rats receiving mangiferin, but non-exposed to TNBS (M10, M30 and M100), no significant changes of all studied parameters were observed in comparison to the control group (group K). Rats exposed to TNBS (group C) developed colitis leading to body weight loss, colon mass index increase as well as macro- and microscopic damage in intestinal tissues compared to the control group. The inflamed colon of rats with TNBS-induced colitis demonstrated mucosal hyperemia, edema, increased mural thickness and distortion of crypts. Additionally, TNBS-induced colitis involved epithelial layer damage, depletion of goblet cells with insufficient production of mucus and a transmural infiltration of inflammatory cells. The mechanism of TNBS-induced intestinal inflammation is likely associated with accumulation of inflammatory cells in the mucosa and impairment of the epithelial monolayer on the mucosal intestinal surface, reflected by an excessive production of pro-inflammatory cytokines and induction of oxidative damage. In the present study, the inflammatory changes in colon tissues caused by TNBS administration were associated with increased TNF-α, IL-17, MDA expression and decreased SOD activity compared to the control group.

Based on the macroscopic, histological and biochemical results obtained for mangiferin pretreated groups with subsequent experimental colitis (CM10, CM30 and CM100), it can be concluded that mangiferin alleviates the course of experimental IBD. Mangiferin at doses of 30 and 100 mg/kg, but not at the dose of 10 mg/kg, reduced the intensity of experimental colitis in rats. Mangiferin at the dose of 100 mg/kg reduced tissue damage caused by TNBS to a greater extent than at the dose of 30 mg/kg, indicating that the protective effect of mangiferin against colon tissue injury is dose dependent. There was no statistically significant difference in measured parameters between control animals and those receiving mangiferin at the highest dose (100 mg/kg) and TNBS. That suggests that mangiferin at the highest dose not only reduces, but also prevents the development of colitis. Additionally, a decrease of the microscopic damage score accompanied by decreased TNF-α, IL-17 and MDA concentrations and increased SOD activity demonstrates the existence of a significant correlation between the degree of severity of microscopic injury and the anti-inflammatory and antioxidant action of mangiferin. Our results demonstrated that all studied doses of mangiferin exerted the antioxidant activity, but the anti-inflammatory effect is evident only at the highest dose. Tissue damage was the least pronounced when mangiferin was used at the highest dose, indicating that the antioxidant activity is insufficient to alleviate the course of experimental inflammatory bowel disease and that the anti-inflammatory activity of mangiferin plays a pivotal role in the mechanism of protective action of the compound.

TNF-α and IL-17 play an important role both in the pathogenesis of IBD and in experimental TNBS-induced colitis. Both cytokines participate in the development of intestinal inflammation ensuring regulation of proliferation, maturation, migration of inflammatory cells and their subsequent activation, enhancement of maturation of dendritic cells and increasing expression of matrix metalloproteases, chemokines and other pro-inflammatory cytokines (Shen and Durum 2010; Strober and Fuss 2011). Thus, a decrease of infiltration and activation of lymphocytes and a subsequent decrease of production of pro-inflammatory cytokines should contribute to reduction of intensity of inflammatory response. That kind of action has been demonstrated in this study and it is in line with targets of modern biological pharmacotherapy. In this study a trend was observed for lower secretion of TNF-α and IL-17 from intestinal tissue in animals pre-treated with mangiferin before the TNBS administration, compared to animals receiving only TNBS. TNF-α and IL-17 concentrations were lower when mangiferin was used at the dose of 100 mg/kg. Mangiferin (30 and 100 mg/kg) decreased size of ulcerated and inflamed areas, restored the epithelial layer, augmented amount of goblet cells and acid glycoproteins (mainly sialomucins) and improved adherents and tight junctions leading to appropriate cover of mucus layer over the intestinal epithelium. Together, those results demonstrate that mangiferin improves integrity of the intestinal mucosal epithelial barrier. Restoration of the intestinal barrier, separation of the gut lumen from the immune system of lamina propria and reduced infiltration of granulocytes, macrophages and lymphocytes into the lamina propria indicate that mangiferin decreases the excessive and uncontrolled antigenic stimulation of non-specific and specific immune responses and inhibits development of inflammation in the intestinal wall.

It is known that overproduction of reactive oxygen species (ROS) in intestinal mucosal cells induces the immune response leading to the injury of intestinal epithelial cells, disruption of integrity of the intestinal barrier and initiation of the intestinal inflammation. One of the most important processes involving free radicals is lipid peroxidation associated with oversynthesis of MDA (Pavlick et al. 2002). Our study demonstrated that mangiferin at all studied doses decreased the MDA level in colon tissues that was significantly increased after the administration of TNBS. It suggests an important antioxidant activity of mangiferin. One of several elements of the cell defense system is superoxide dismutase—the enzyme that catalyzes the reaction of dismutation of the superoxide anion. In the present paper only mangiferin administered at the highest dose increased SOD activity in TNBS-induced inflamed colon tissue, which suggests that the enzyme may be a less sensitive predictor of oxidative stress compared to MDA.

The anti-inflammatory action of mangiferin demonstrated in this paper is in line with results reported by other authors. Márquez et al. (2010) demonstrated a protective effect of the *Mangiferae indica* extract (containing mangiferin) on colonic tissues in experimental colitis caused by dextran sulfate sodium. Tested extract decreased serum IL-6 and TNF-α concentrations and reduced TNF-α and TNF-α type 2 receptor expression in colonic tissues. Recently, some studies using pure mangiferin have been also published. However, all of them used the murine model of colitis. Jeong et al. (2014) demonstrated that in TNBS-induced intestinal inflammation in mice mangiferin decreased the degree of colon shortening and reduced macroscopic lesions. Furthermore, mangiferin diminished expression of TNF-α, IL-1β, IL-6, inhibited IL-1R-associated kinase phosphorylation and activation of the nuclear factor kB. Similarly, the murine TNBS model of colitis was used by Lim et al. (2016) who demonstrated that TNBS-induced increase in TNF-α and IL-17 levels were reduced by pre-treatment with mangiferin. Two more papers reported the murine colitis model but the disease was induced by a different irritating factor—dextran sodium sulfate (DSS) in drinking water. Dou et al. (2014) demonstrated mainly the anti-inflammatory action of mangiferin (TNF-α, NF-κB, iNOS), whereas Somani et al. (2016) focused mainly on oxidative stress parameters (CAT, GSH, SOD, MDA, MPO), but also revealed some anti-inflammatory effects of mangiferin.

To our best knowledge, our study is the first to demonstrate the protective effect of pure mangiferin in experimentally induced colitis in rats. Besides some basic parameters of inflammation and oxidative stress that had been also studied by other researchers, we broadened our study by a more detailed histopathological evaluation. Besides the standard hematoxylin-eosin staining, demonstrating some typical micropathological damage to the colon tissue, we used alcian blue and Giemsa staining. The first reveals the colonic mucus layer with goblet cells producing sialomucins, whereas Giemsa staining allows to assess the magnitude of inflammatory cell infiltration to the colonic tissue. Both these processes play a very important role in the pathogenesis and determination of the degree of damage in colitis. Based on these findings we may state that mangiferin increases the integrity of epithelial layer and significantly decreases colonic infiltration by inflammatory cells.

Restoration of integrity of the mucosal epithelial barrier, decreased production of pro-inflammatory cytokines, prevention of lipid peroxidation and increased activity of antioxidant enzymes could be considered as mechanisms involved in the beneficial effect exerted by mangiferin. Considering findings from our and other studies it can be assumed that mangiferin alleviates the course of experimental colitis in rats and mice. Explanation of detailed molecular mechanisms involved in the beneficial effect of mangiferin requires further investigations of, e.g., the impact of mangiferin on the expression of protective proteins (e.g., trefoil factor 3, mucins), transcription factors, caspases or mitogen-activated protein kinases engaged in the activation of the inflammatory cascade in IBD.

## Conclusions

In conclusion, mangiferin alleviates the colonic inflammatory changes in TNBS-induced experimental colitis in rats. Pre-treatment with mangiferin at the doses of 30 and 100 mg/kg diminishes macro- and microscopic colonic damage and leads to reduced MDA concentration. Moreover, pre-treatment with mangiferin at the dose of 100 mg/kg decreases TNF-α and IL-17 concentrations and increases SOD activity. The antioxidant activity is evident after administration of mangiferin at all studied doses and the anti-inflammatory effect is observed only after administration of mangiferin at the highest dose (100 mg/g). In parallel, the least pronounced injury of colon tissue develops following administration of mangiferin at the highest dose. It may, therefore, be concluded that the crucial mechanism underlying the protective impact of mangiferin observed in this report is associated with the compound’s anti-inflammatory action and that its antioxidant action is of an accessory character.

## Abbreviations

IBD: Inflammatory bowel disease
CD: Crohn’s disease
UC: Ulcerative colitis
TNF α: Tumor necrosis factor α
IL: Interleukin
TNBS: Trinitrobenzensulfonic acid
MDA: Malondialdehyde
SOD: Superoxide dismutase
ROS: Reactive oxygen species
GPx: Glutathione peroxidase
CAT: Catalase
GR: Glutathione reductase
